# Examining the growing challenge: Prevalence of diabetes in young adults (Review)

**DOI:** 10.3892/mi.2024.201

**Published:** 2024-11-06

**Authors:** Ghulam Murtaza, Samavia Riaz, Maria Zafar, Muhammad Ahsan Raza, Imdad Kaleem, Hadia Imran, Aryam T. Al-Harbi, Ali Sabouri, Talha Asim Niaz, Shahid Bashir

**Affiliations:** 1 Department of Zoology, University of Gujrat, Gujrat, Punjab 50700, Pakistan; 2 Government Graduate College (boys), Gujranwala, Punjab 50250, Pakistan; 3 Department of Biosciences, COMSATS University Islamabad (CUI), Islamabad 45550, Pakistan; 4 Department of Medicine, College of Medicine, Imam Abdulrahman Bin Faisal University, Dammam 34212, Kingdom of Saudi Arabia; 5 Department of Oncology, Mount Vernon Cancer Centre, East and North Hertfordshire NHS Trust, Northwood HA6 2RN, UK; 6 Faculty of Medicine, University of Southampton, Southampton, SO17 1BJ, UK; 7 Department of Neuroscience, Neuroscience Center, King Fahad Specialist Hospital Dammam, Dammam 31444, Kingdom of Saudi Arabia

**Keywords:** diabetes, prevalence, prevention, management, global health

## Abstract

Diabetes is rapidly spreading worldwide, affecting millions of individuals. Therefore, it is crucial to have a comprehensive understanding of its complications. The present review discusses the complex subject of diabetes, including the type 1 and type 2 variants. Geographical and population differences highlight the importance of targeted therapies and personalized management strategies. Ongoing research aims to identify the causes and treatment strategies for this disease. Preventive interventions, lifestyle changes and public awareness campaigns are all vital components of diabetes management. Collaboration between the general public and health departments is essential for effective prevention. Early intervention and global management strategies are necessary to reduce the significant impact on healthcare systems. A comprehensive plan from health care departments is required to address the issues caused by diabetes and minimize its effects on individuals and communities worldwide. The present review outlines specific measures which can be used to combat the spread of diabetes for a healthier future world.

## 1. Introduction

Diabetes also known as diabetes mellitus (DM) has become a critical global public health concern, characterized by an abnormal glucose metabolism that can lead to severe complications in various organ systems (1). Diabetic patients have high blood sugar levels due to deficiency of insulin and resistance or impaired insulin secretion. Diabetes is primarily comprised of two types, type 1 and type 2 diabetes. Type 1 diabetes is considered an autoimmune condition that results from the destruction of insulin-producing β-cells, causing the body to be unable to produce insulin. This type of diabetes typically develops in childhood or adolescence (2). Symptoms include rapid onset, increased thirst and urination and weight loss. It has various effects on patients, including dependence on insulin injections, an increased risk of hypoglycemia (low blood sugar) and potential long-term complications such as kidney damage, nerve damage and vision-related isues. It can be managed with insulin therapy and lifestyle modifications (2).

Type 2 diabetes is a metabolic condition caused by insulin resistance and decreased insulin secretion (3). It typically develops in adulthood and is associated with obesity and physical inactivity. Symptoms include gradual onset, increased thirst and urination, and fatigue. It can lead to an increased risk of developing cardiovascular diseases, complications during pregnancy and insulin dependence (3). It can be managed through lifestyle changes (such as a healthy diet and exercise), medications and insulin therapy (3,4).

It is estimated that ~422 million individuals are living with diabetes worldwide with the majority belonging to low-and middle-income countries. Diabetes causes 1.5 million deaths each year, and the prevalence and number of diabetic cases have been rapidly increasing globally (5). The prevalence of DM among adults has been on the rise over the past few decades. In 1964, an estimated 30 million individuals had diabetes (6). By the year 2000, the World Health Organization (WHO) estimated that 171 million individuals had diabetes, projecting this number to rise to 366 million by the year 2030(7). Similarly, the International Diabetes Federation (IDF) estimated 151 million cases of DM worldwide in 2000(8), which aligned closely with the WHO estimation. Furthermore, the IDF estimated 194 million cases of diabetes in 2003(9), 285 million in 2009(10), 382 million in 2013(11), 463 million in 2019(12) and 571 million in 2021. These numbers are projected to increase to 643 million by 2030 and 783 million by 2045(13). The report of the IDF also states that type 1 diabetes affected 8.75 million individuals worldwide in 2022, with 1.52 million of these individuals being <20 years of age (14).

## 2. Global variations and emerging trends in the prevalence of diabetes

A marked increase in the prevalence of DM has been observed worldwide, in both urban and rural areas, affecting individuals of all age groups, particularly young adults (15). Accurate estimates and projections of DM prevalence are necessary for planning, executing and monitoring treatment and prevention strategies. In 2014, the average diabetes prevalence was 8.5% and the diabetic population is projected to reach 642 million by 2040, up from 422 million (15).

Notably, diabetes and its associated complications are leading causes of mortality. From 1990 to 2010, the global ranking of the disease surged from 15 to 9th place, resulting in a substantial 92.7% increase in burden, indicating a steady rise in incidence and signaling a concerning trend (16). The US has experienced a rapid escalation in diabetes cases over the past two decades. According to the National Health Interview Survey (NHIS) data spanning from 1980 to 2005, the age-adjusted occurrence of diagnosed diabetes remained relatively constant at 3.0% from 1980 to 1990 before witnessing an upward trajectory. In 1990, the age-adjusted prevalence was approximately 2.9%, rising to 4.5% in 2000 and 5.3% in 2005 showing a continuous decline in the quality of diet and life with the revolution (17). The analysis of NHIS data from 1997 to 2005 reveals a consistently higher burden of diabetes among adults aged 25 years (17). Additionally, African Americans exhibit higher age-adjusted rates of diagnosed diabetes compared to Americans. According to current estimates, 3.2 million African Americans are affected. Projections indicate a tripling of diabetes cases by the year 2050, underscoring the critical need for prevention and management approaches (15).

Diabetes has emerged as a global health concern, affecting 8.5% of individuals aged ≥18 years in 2014. This represents a significant increase over the past three decades, particularly in developing Asian countries (15). The Eastern Mediterranean Area reports the highest average occurrence of diabetes in adults, reaching 13.7% in 2014, surpassing rates in other WHO regions (15). Poland stands out with a notably high diabetes rate, affecting ~8% of the population (18). This prevalence is significantly higher than the EU average of 6.3%, and projections indicate a rise to 11% by 2040(18). As regards Iran, the prevalence of diabetes among individuals aged 25 to 70 years was 11.9% in 2011, signifying a 35% increase from 2005. Projections estimate that by 2030, ~9.2 million Iranians will be diagnosed with diabetes (19). This information underscores the global impact of diabetes, with varying prevalence rates across regions and nations. The identification of these trends is critical for developing targeted interventions and public health initiatives to address the rising burden of diabetes worldwide. The projected increase in diabetes cases is alarming, as it will strain healthcare systems, increase healthcare costs, exacerbate health disparities and affect the economic productivity and quality of life of populations.

## 3. Environmental factors involved in the progression of diabetes

Environmental factors such as health services, safety, amenities, area conditions, public transport and green space play a crucial role in the transition from latent autoimmunity to clinically evident illness (20). This transition can be influenced by improved living conditions that decrease vulnerability to certain pathogens, ultimately leading to heightened autoimmune responses. Individuals who reside in walkable areas are more likely to walk more frequently, thus reducing their risk of obesity (20).

## 4. Geographical variations in the prevalence of type 1 diabetes

There are notable geographical variations in the pattern of type 1 diabetes. It is least common in rural areas of developing countries, usually average in developed countries, and most common among several ethnic groups, particularly those assimilating into Western lifestyles. Gene-environment interactions play a crucial role in the prevalence of diabetes. The incidence or prevalence of type 1 diabetes varies across different parts of China (21). In some regions, the rate of type 1 diabetes is 12-fold higher or lower compared to other regions (21). This significant area variation is due to multiple factors, such as genetic diversity, environmental influences, healthcare access and lifestyle differences across various parts of the country (22,23). These studies highlighted that type 1 diabetes is not uniformly distributed and has higher and lower incidence across different parts of China (21-23).

Genetic predisposition to insulin resistance is exacerbated by environmental factors, such as a sedentary lifestyle and a high-calorie diet with genes, such as transcription factor 7 like 2, potassium inwardly rectifying channel subfamily J member 11 and peroxisome proliferator-activated receptor (PPAR)G contributing to this interaction (24). Epigenetic modifications, influenced by maternal nutrition and stress, affect gene expression and increase the risk of developing diabetes, with genes such as insulin induced gene 2 and PPARγ being affected. These interactions support the ‘thrifty genotype’ theory, which suggests that genetic variants linked with insulin resistance, as well as diabetes provide an evolutionary advantage in ancestral environments, such as during famines, but become detrimental in modern environments characterized by an abundance of resources (24,25). These findings highlight the complex interplay among genetic and environmental factors contributing to the risk of developing diabetes (25).

Individuals moving from areas where type 2 diabetes is less prevalent to Western countries are at a greater risk of developing type 2 diabetes. Notably, compared to European Caucasian populations, the prevalence of diabetes in the UK is 4-6-fold times higher among African Caribbeans and South Asians (26). This highlights the influence of genetic and environmental variables on the incidence of diabetes, illuminating the intricate association between lifestyle, geography and the risk of developing diabetes.

## 5. Lifestyle and genetic predisposition: Implications for Asia's emerging health trends

According to the UN, >60% of the world's youth reside in the Asia-Pacific region (27). Additionally, a 2011 survey found that 65% of the Indian population is <35 years of age. This youthful population in Asia comprising 65% of the total population is rapidly adapting to new lifestyles that were previously unfamiliar to them (26,28). These changes include adopting a more Westernized diet, spending an increased amount of time in front of a screen, engaging in decreased levels of physical activity, urbanization, exposure to environmental pollutants, and the increased consumption of processed and fast food. These lifestyle changes pose a significant risk for the development of diabetes and its related complications. Not only does this evolving lifestyle affect individual health, but it also places a significant financial burden on developing countries.

Concerns have been raised about the lack of physical activity among young individuals globally, where 78% of boys and 84% of girls do not meet the minimum exercise requirements for their age. This has led to a noticeable sex disparity with females being more overweight than males (29). A study conducted out in an Indian urban slum revealed a high incidence of metabolic disorders, such as diabetes and obesity among middle-aged individuals, particularly women in this economically disadvantaged group (30). The significant impact that lifestyle changes have on health outcomes underscores the need for targeted interventions and educational campaigns to address the new health challenges faced these communities.

## 6. Types of diabetes and differences between them

### Type 1 diabetes

Type 1 diabetes, also known as insulin-dependent diabetes, is an autoimmune condition that typically presents in childhood or adolescence, but can also affect young adults. According to the American Diabetes Association, an estimated 1.6 million Americans have type 1 diabetes, with a significant number of diagnosis occurring during adolescence or early adulthood (1). The prevalence of type 1 diabetes varies among countries and regions, with an increasing frequency observed in certain western countries. The unique characteristics of type 1 diabetes, such as its sudden onset, and the necessity for immediate medical attention contribute to the accurate identification of new cases (31). This underscores the importance of timely diagnosis and intervention in managing type 1 diabetes, particularly given its onset during crucial developmental stages in individuals.

### Geographical variations in the incidence of type 1 diabetes in different age groups

There is significant geographical variation in the incidence of type 1 diabetes. In individuals aged 0-19 years the incidence rates have been found to be 39 per 100,000 individuals in Northern Europe, 20.97 per 100,000 individuals in North America, 10.1 per 100,000 individuals in Northern Africa, and 0.5 per 100,000 individuals in Western Africa (31). Although type 1 diabetes can develop at any age, the majority of cases involve individuals between the ages of newborn and 14 years. For instance, the highest incidence in children aged 0-4 years was found in Northern Africa (31.11 per 100,000 individuals), followed by Northern Europe, with an incidence of 21.54 per 100,000 individuals, and the lowest in Eastern Asia, Western Asia, Southern Asia and Eastern African countries (<5 per 100,000 individuals) (31). In children aged 10-14 years, the highest incidence was found in Northern Europe (41.48 per 100,000 individuals) and the lowest in Southern Asia (1.99 per 100,000 individuals). Furthermore, in children aged 15-19 years, the highest incidence was in Northern America, with a rate of 17.68 per 100,000 individuals, while the lowest was in African countries with an incidence rate of 1.07 per 100,000 individuals. The incidence rate of type 1 DM was the highest (18.02 per 100,000 individuals) in children aged 10-14 years (31). This regional heterogeneity highlights the complex interplay between environmental and genetic variables that determine the prevalence of type 1 diabetes in various parts of the world.

### Sex, income and ethnicity differences in type 1 diabetes

The occurrence of diabetes may vary according to the sex and income of individuals. The incidence rates of type 1 diabetes differ among males and females in various regions of the world (31). For example, in males from Northern Africa, Australia/New Zealand, North America and Southern Asia, the incidence rates were found to be 40.51 per 100,000 individuals, 23.72 per 100,000 individuals, 18.57 per 100,000 individuals and 1.01 per 100,000 individuals, respectively (31). Among females from Northern Africa, Australia/New Zealand, North America and Southern Asia, the incidence rates were 36.49 per 100,000 individuals, 22.33 per 100,000 individuals, 17.50 per 100,000 individuals and 1.05 per 100,000 individuals, respectively (31). As regards the association of income with diabetes, the incidence rates of type 1 diabetes were 7.89 per 100,000 individuals, 0.87 per 100,000 individuals, 0.57 per 100,000 individuals and 0.19 per 100,000 individuals in high-income, upper-middle-income, lower middle income and low-income individuals, respectively (31). Considering ethnicity, different ethnic groups have distinct genetic backgrounds, lifestyles and environmental exposures that influence the risk of type 1 diabetes. Among 7,000 US males and females aged 45 to 84 years with a given waist circumference, the highest incidence of diabetes was reported in Chinese Americans followed by individuals of Hispanic, African and European ancestry (32). Understanding different patterns (geography, age, sex, ethnicity etc.) related to DM is critical for tailoring effective interventions and healthcare policies to address specific demographic groups who are at a greater risk of developing type 1 diabetes.

### Temporal variations in type 1 diabetes

The average annual global growth rate of type 1 diabetes is 2.8 to 3.0% (DIAMOND Project Group, 2006), with Europe experiencing a documented growth rate of 3.9%. Notably, low-incidence countries exhibit a larger relative growth (33). The age group of 0 to 4 years has the largest significant growth. Additionally, there is seasonal fluctuation in the prevalence of type 1 diabetes, with the highest rates observed in the autumn and winter (33). Understanding the temporal trends and fluctuations in type 1 diabetes prevalence is essential for implementing timely and effective public health measures to address the increasing burden of the disease (33).

### Etiological factors for type 1 diabetes

Environmental factors and genetic predispositions, in combination, cause type 1 diabetes. The transition from islet autoimmunity to a clinically evident disease is largely influenced by environmental factors. Improving living conditions may play a role by reducing microbial exposure and enhancing autoimmune responses. Factors such as community interaction, illness, pollutants and dietary choices, such as limited breastfeeding and the delayed introduction of cow's milk have all been implicated in various studies (10,12). A potential contributing factors is inadequate levels of vitamin D (12). Additionally, some theories suggest that omega-3 fatty acids may play a role in the onset of type 1 diabetes (14). Understanding the complex interaction between genetics and environmental factors is crucial for identifying preventive measures and uncovering the root causes of type 1 diabetes.

### Type 2 diabetes

Young individuals and teenagers are being diagnosed with type 2 diabetes at an increasing rate (34). This development is linked to poor nutrition and a sedentary lifestyle. Factors, such as ethnicity, race, family history and socioeconomic levels may influence the prevalence of this condition among young adults (3,4,34). According to the centers for Disease Control and Prevention (CDC), type 2 diabetes is entirely associated with being overweight and its incidence is increasing among young adults. Countries and regions differ in the percentage of the diabetic population, and public health programs, healthcare access and education all have a significant impact on these differences (34). To effectively avoid and manage type 2 diabetes in adolescents, it is necessary to recognize and address these complex aspects.

### Differences in the prevalence of type 2 diabetes as regards geographical location, ethnicity, age and sex

Type 2 diabetes exhibits marked geographical variations, although with different trends compared with type 1 diabetes (35). It is less prevalent in developing countries, such as rural regions of Nigeria and India, and more common in developed countries such as the US and the UK. In 2019, there was an increase in the incidence of type 2 diabetes in terms of age-standardized prevalence rate, age-standardized mortality rate, age-standardized incidence rate and age-standardized disability-adjusted life years in the low-middle socio-demographic index areas of the world (35). It is more prevalent in certain ethnic groups, such as African American and Hispanics. Of note, those with the highest prevalence of type 2 diabetes also tend be obese. Genetic susceptibility to obesity is considered to have been advantageous in times of scarcity, but detrimental in times periods of abundance, potentially continuing through natural selection (25).

Individuals who migrate to Western countries from regions with a low prevalence have a higher risk of developing type 2 diabetes. For example, compared to Caucasian European populations, South Asians and Africans in the UK exhibit 4-6-fold higher rates of diabetes (26). These disparities underscore the complex interplay between lifestyle, environmental and genetic factors that affect the occurrence of type 2 diabetes in different geographic locations, demographics and ethnic groups. Understanding these differences is essential in tailoring effective healthcare and public health strategies (26).

### Prevalence and temporal variation in type 2 diabetes

Research utilizing serial glucose tolerance testing indicates an annual prevalence of ~7 per 1,000 in Western populations, particularly in Europeans (36). Individuals with impaired glucose tolerance (IGT) exhibit an ~10-fold higher incidence of diabetes compared with those with normal glucose tolerance (37). Additionally, hyperglycemic conditions such as gestational diabetes increase an individual's risk of developing diabetes later in life.

The global diabetic population is projected to increase from 382 million in 2013 to 592 million in 2035(18). Developing nations are expected to exhibit a sharper increase, particularly in adopting Western lifestyles. Dabelea *et al* (38) reported that the prevalence of type 2 diabetes in the US increased from 0.34 per 1,000 to 0.46 per 1,000 individuals between 2001 and 2009, with American Indian, African and Hispanic children exhibiting higher rates than Caucasian adolescents. Globally, there has been an increase in the prevalence of type 2 diabetes from 2.8% in 2000 to 9.3% in 2019, with a particularly high prevalence among younger adults (20-39 years) and higher rates in urban areas (10.4%) compared with rural areas (7.2%). Taking into account these temporal trends, the prevalence is expected to reach 11.2% by 2030 and 13.8% by 2050, with a projected to increase of 69% in developing countries compared to 20% in developed countries (38). These findings underscore a significant temporal fluctuation and concerning patterns in the rate of type 2 diabetes globally and within specific populations. Urgent action is required to implement preventive measures to address the rising prevalence, focus on high-risk populations, promotes lifestyle modifications, and improve screening and timely intervention. Addressing these trends and implications is crucial for effective public health interventions to mitigate the growing burden of type 2 diabetes (38).

### Etiological factors for type 2 diabetes

Insulin resistance and insulin secretory insufficiency are the most prevalent pathophysiological abnormalities linked to type 2 diabetes (4). The three main risk factors are age, a family history of obesity and physical inactivity. Potential nutritional risk factors include consuming large amounts of red or processed meat (38), sweetened beverages (39), and fruits and vegetables (40). Dietary patterns can also play a role in reducing the risk. Novel strategies that utilize quantifiable dietary indicators can advance the understanding of the connection between diabetes and nutrition.

Despite the substantial genetic component of type 2 diabetes, >60 variants have recently been linked to an increased risk of developing the disease; however, the individual effects of these genetic variations are minimal (41). When combined with genetic scores, the identified genes have a minimal contribution to predicting the risk of developing diabetes. Incorporating lifestyle and clinical characteristics into phenotype-based risk models enhances their ability to predict diabetes. Adding genotypic evidence increases prediction accuracy by 5 to 10%. Although genetic variants offer valuable insight into the etiology and molecular processes of diabetes, their predictive power however, remains limited (42).

## 7. Risk factors for diabetes in young adults

**Figure 1:**
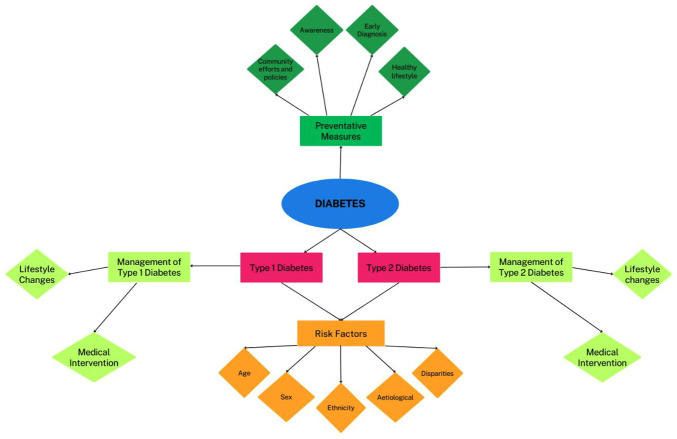
Schematic diagram depicting the types of diabetes, and risk factors and management strategies.

The risk factors for diabetes in young adults are multifaceted and influenced by various elements. Lifestyle variables, including poor dietary habits and physical inactivity, play a substantial role, contributing to an increased risk of obesity (Fig. 1) (43). Lifestyle factors such as diet (consuming high-sugar, high fat and high-sodium foods, and processed meals) and physical inactivity (sedentary lifestyle, prolonged periods of sitting) play a crucial role in increasing the risk of developing diabetes, particularly among young adults (43). These factors increase the risk of developing diabetes by causing insulin resistance, the dysfunction of pancreatic β-cells, and chronic inflammation (43). Moreover, the risk increases by 30-50% with the consumption of unhealthy foods, whereas obesity alone is associated with a 2-5-fold increased risk. However, diabetes can be prevented by maintaining a balanced diet, engaging in physical activity, weight management, reducing stress, and receiving adequate sleep (43). Diabetes risk is also greatly influenced by socioeconomic situations, family history and genetics. Type 1 diabetes can develop at any age, although it is more common in children, adolescents and young adults, particularly in those who have prediabetes or are overweight (38). Individuals with a sibling, parent, or relative with the disease are at an increased risk. Diabetes in general can be influenced by family history, and individuals with a family history of type 1 diabetes may be subjected to an autoantibody test, as type 1 diabetes occurs commonly in those with autoantibodies. Furthermore, location and environmental factors may influence the development of type 1 diabetes, while ethnicity may potentially elevate the threat of the occurrence of type 2 diabetes. Understanding and addressing these diverse risk factors are crucial for developing targeted prevention strategies and early interventions for diabetes in young adults (43).

## 8. Health consequences of diabetes

Diabetes can lead to a variety of health issues, significantly affecting different organ systems and resulting in severe consequences. The increasing prevalence of diabetes has a significant impact on healthcare systems and economies. Complications related to diabetes include cardiovascular diseases, renal diseases, blindness, neuropathy, as well as additional dental, visual and mental health concerns (4). Persistent issues include damage to the blood arteries, which can lead to heart attacks and strokes (4). Mild cognitive dysfunction is also a recognized consequence of diabetes and its management (44). Over time, diabetes can affect multiple organ systems in the body, causing severe consequences.

Diabetes-related complications are broadly classified into microvascular and microvascular problems. Microvascular complications include damage to the nervous system (neuropathy), kidneys (nephropathy), and eyes (retinopathy) (1). The latter can result in bruising, lesions, gangrene, and, ultimately, the need for amputation (1). Understanding and addressing these health consequences are crucial for comprehensive diabetes management and prevention strategies.

## 9. Disparities in diabetes

The prevalence of diabetes varies significantly among different regions and populations leading to disparities in healthcare outcomes. The differences in healthcare approaches and available resources for managing diabetes play a crucial role. The condition is rapidly increasing in developed countries (45). Previous studies have shown that socioeconomic status is more relevant in identifying individuals with diabetes compared to non-modifiable risk factors like ethnicity (46,47).

Previous research has consistently shown an increased occurrence of diabetes in economically depressed regions or among individuals of lower socioeconomic status (46). While socioeconomic status is not a biological determinant, it can help identify other proven risk factors for diabetes, such as body mass, exercise, hypertension and gestational diabetes. Using socioeconomic status can help identify target groups for primary and secondary interventions.

There is evidence to suggest that racial and ethnic minorities are more likely to develop diabetes and may receive lower-quality care leading to higher prevalence rates, earlier onset, greater severity, and more complications. Factors contributing to these disparities include socioeconomic barriers (poverty, limited access to healthcare), a shortage of healthcare workers, limited health literacy, and inadequate access to healthy food. This can result in poorer glycemic control, higher chances of diabetes, and lower life expectancy. Racial and ethnic minorities often experience inadequate screenings and follow-ups, poor medication management, and insufficient referrals to specialists.

Addressing these disparities requires a multifaceted approach involving healthcare providers, policy makers and communities, to ensure equitable access to quality diabetes care. Implementing diabetes care interventions can improve health outcomes and reduce racial and ethnic health disparities (46). Additionally, addressing these disparities is essentials for achieving equitable healthcare and improving outcomes for all individuals affected by diabetes.

## 10. Preventive measures for diabetes

Addressing the increasing prevalence of diabetes involves public health initiatives focused on prevention through lifestyle modifications, education and early detection. The following preventive measures are crucial: i) Encouraging healthy food choices such as whole, unprocessed foods, lean protein sources and healthy fats, while avoiding refined carbohydrates, and saturated fats is essential for preventing diabetes. Regular physical activity, involving 150 min of medium-intensity physical activity weekly, and weight management, maintaining a healthy body mass index through sustainable lifestyle changes are also vital components. Together, these factors improve insulin function, regulate blood sugar levels, reduce chronic inflammation, promote overall metabolic health and can considerably decrease the risk of developing diabetes. ii) Regrettably, type 1 diabetes is not yet preventable (47). iii) Active measures can prevent the complications and early mortality associated with type 2 diabetes. These include policies and practices promoting good health for everyone, regardless of their diabetes status. Key measures include proper exercise, a healthy diet, and the management of blood pressure and lipid levels. iv) The early detection of diabetes is crucial, as it identifies high-risk individuals, detects undiagnosed diabetes, enables timely intervention and prevents complications. Basic diagnostics include fasting plasma glucose, oral glucose tolerance test, random blood glucose, and urine glucose. In primary healthcare settings, convenient and cost-effective access, early intervention, patient education, and continuous monitoring are crucial. Diabetes screening and diagnostics should be integrated into primary healthcare settings to ensure timely intervention and optimal management. v) A number low-cost treatments can correct patient outcomes. These include regulating blood pressure and cholesterol to lower the risk of heart attack and other issues; frequent screenings for kidney and eye health to aid in early diagnosis and treatment; and maintaining blood glucose levels through diet, exercise, and medication. vi) Losing 5-10% of one's current weight can significantly contribute to preventing or delaying the onset of diabetes (1).

Communities and healthcare systems can collaborate to lessen the impact of diabetes and improve general public health by supporting these preventative initiatives.

## 11. Medical management of diabetes

Effectively managing diabetes involves a combination of medications, insulin therapy, and self-management training. Some key aspects of medical management for both type 1 and type 2 diabetes are as follows: i) For type 1 diabetes: Insulin treatment is essential, and individuals not receiving it can experience severe health complications. ii) For type 2 diabetes: Insulin may be necessary for a large number of individuals with type 2 diabetes. Insulin is typically injected beneath the skin, and inhaled insulin is another option for some individuals. iii) Those with type 2 diabetes can effectively lower their blood glucose levels with these drugs. For individuals who have type 1 diabetes, these drugs are ineffective (48). iv) This monoclonal antibody may delay the onset of symptoms in certain individuals with type 1 diabetes. It is administered once a day for 14 days and can prevent the emergence of symptoms for ~2 years (49). v) These medications are used to manage blood pressure and are recommended for individuals with diabetes, particularly those with high blood pressure or persistent renal disease (50). vi) To reduce the risk of heart disease, aspirin may be advised for diabetics with high blood pressure or chronic renal illness. vii) Individuals with type 1 diabetes may be eligible for an islet cell transplant, offering a potential therapeutic option (51).

Medical management should be personalized based on the type of diabetes, individual health conditions and other relevant factors. The effective management of diabetes requires lifestyle changes, regular monitoring and adherence to prescribed drug schedules.

## 12. Research and awareness in diabetes

Continued research in diabetes, including its causes, prevention and therapy, is crucial for advancing the understanding and improving patient outcomes. Promoting awareness of diabetes and its risk factors plays a pivotal role in early detection and effective management. Some key points regarding research and awareness are as follows: i) Diabetes research is a dynamic field, exploring various aspects such as causes, prevention strategies and therapeutic interventions. Continued efforts in research are essential for advancing the current knowledge and developing innovative treatments (52-54). ii) Enhancing the understanding of diabetes and its hazardous factors is vital for early detection and proactive management. The consistent monitoring of public knowledge about diabetes is essential for implementing efficient informative and precautionary measures (55). iii) Patients with diabetes are encouraged to actively participate in their disease care. High levels of engagement and adherence to treatment plans can significantly improve the value of life for individuals with diabetes and help delay long-term consequences. iv) Diabetes screening programs, such as a general screening programs, are valuable for early detection (52,53). These initiatives increase public awareness and the comprehension of diabetes, reducing diagnostic gaps and preventing long-term complications for those who have the disease (43).

Study initiatives and raising public awareness are interconnected components of comprehensive diabetes care, influencing the development prevention, early detection and management of the condition (55).

## 13. Conclusion and future perspectives

Diabetes is a pressing worldwide public health issue with a variety of causes and effects. The increasing global prevalence of diabetes presents significant challenges to healthcare systems worldwide. It calls for a deeper understanding of the risk factors and causes of the disease, as well as effective management strategies for its control. Preventative measures, health management, research and awareness are all key components of comprehensive approaches that are required to address the global rise in the prevalence of diabetes and improve its results for its patients. Improving public health requires determining a collective approach and reducing biases.

## Data Availability

Not applicable.

## References

[1] (2006). Diagnosis and classification of diabetes mellitus. Diabetes Care.

[2] Lucier J, Dulebohn SC https://www.ncbi.nlm.nih.gov/books/NBK507713/.

[3] Goyal R, Singhal M, Jialal I https://www.ncbi.nlm.nih.gov/books/NBK513253/.

[4] Banday MZ, Sameer AS, Nissar S (2020). Pathophysiology of diabetes: An overview. Avicenna J Med.

[5] https://www.who.int/health-topics/diabetes?gad_source=1&gclid=EAIaIQobChMIuKKXgvWFiAMVszYGAB3vNQJFEAAYASAAEgKTq_D_BwE#tab=tab_1.

[6] Entmacher PS, Marks HH (1965). Diabetes in 1964; a world survey. Diabetes.

[7] Wild S, Roglic G, Green A, Sicree R, King H (2004). Global prevalence of diabetes: Estimates for the year 2000 and projections for 2030. Diabetes Care.

[13] Magliano DJ, Boyko EJ https://www.ncbi.nlm.nih.gov/books/NBK581934/.

[15] https://www.who.int/health-topics/diabetes#tab=tab_1.

[16] Lozano R, Naghavi M, Foreman K, Lim S, Shibuya K, Aboyans V, Abraham J, Adair T, Aggarwal R, Ahn SY (2012). Global and regional mortality from 235 causes of death for 20 age groups in 1990 and 2010: A systematic analysis for the global burden of disease study 2010. Lancet.

[17] DeFronzo RA, Ferrannini E, Groop L, Henry RR, Herman WH, Holst JJ, Hu FB, Kahn CR, Raz I, Shulman GI (2015). Type 2 diabetes mellitus. Nat Rev Dis Primers.

[18] Saeedi P, Petersohn I, Salpea P, Malanda B, Karuranga S, Unwin N, Colagiuri S, Guariguata L, Motala AA, Ogurtsova K (2019). Global and regional diabetes prevalence estimates for 2019 and projections for 2030 and 2045: Results from the international diabetes federation diabetes atlas, 9th edition. Diabetes Res Clin Pract.

[19] Mirzaei M, Rahmaninan M, Mirzaei M, Nadjarzadeh A, Dehghani Tafti AA (2020). Epidemiology of diabetes mellitus, pre-diabetes, undiagnosed and uncontrolled diabetes in Central Iran: Results from Yazd health study. BMC Public Health.

[20] Dendup T, Feng X, Clingan S, Astell-Burt T (2018). Environmental risk factors for developing type 2 diabetes mellitus: A systematic review. Int J Environ Res Public Health.

[21] Weng J, Zhou Z, Guo L, Zhu D, Ji L, Luo X, Mu Y, Jia W (2018). Incidence of type 1 diabetes in China, 2010-13: Population based study. BMJ.

[22] Wu NW, Lyu XF, An ZM, Li SY (2024). Adiposity in Chinese people with type 1 diabetes. World J Diabetes.

[23] Li J, Shi Q, Gao Q, Pan XF, Zhao L, He Y, Tian H, Zhu Z, Li S (2022). Obesity pandemic in China: Epidemiology, burden, challenges, and opportunities. Chin Med J (Engl).

[24] Tremblay J, Hamet P (2019). Environmental and genetic contributions to diabetes. Metabolism.

[25] Southam L, Soranzo N, Montgomery SB, Frayling TM, McCarthy MI, Barroso I, Zeggini E (2009). Is the thrifty genotype hypothesis supported by evidence based on confirmed type 2 diabetes- and obesity-susceptibility variants?. Diabetologia.

[26] Forouhi NG, Luan J, Hennings S, Wareham NJ (2007). Incidence of type 2 diabetes in England and its association with baseline impaired fasting glucose: The Ely study 1990-2000. Diabet Med.

[27] http://http://121.171.142.202/announcement/youth-and-mental-health-asiapacific.

[28] Muscogiuri G, Verde L, Vetrani C, Barrea L, Savastano S, Colao A (2024). Obesity: A gender-view. J Endocrinol Invest.

[29] Hallal PC, Andersen LB, Bull FC, Guthold R, Haskell W, Ekelund U (2012). Global physical activity levels: Surveillance progress, pitfalls, and prospects. Lancet.

[30] Misra A, Pandey R, Devi JR, Sharma R, Vikram N, Khanna N (2001). High prevalence of diabetes, obesity and dyslipidaemia in urban slum population in northern India. Int J Obes Relat Metab Disord.

[31] Gomber A, Ward ZJ, Ross C, Owais M, Mita C, Yeh JM, Reddy CL, Atun R (2022). Variation in the incidence of type 1 diabetes mellitus in children and adolescents by world region and country income group: A scoping review. PLoS Glob Public Health.

[32] Lutsey PL, Pereira MA, Bertoni AG, Kandula NR, Jacobs DR Jr (2010). Interactions between race/ethnicity and anthropometry in risk of incident diabetes: The multi-ethnic study of atherosclerosis. Am J Epidemiol.

[33] Stahl-Pehe A, Kamrath C, Prinz N, Kapellen T, Menzel U, Kordonouri O, Schwab KO, Bechtold-Dalla Pozza S, Rosenbauer J, Holl RW (2022). Prevalence of type 1 and type 2 diabetes in children and adolescents in Germany from 2002 to 2020: A study based on electronic health record data from the DPV registry. J Diabetes.

[34] Pinhas-Hamiel O, Zeitler P http://MDText.com.

[35] Liese AD, Lawson A, Song HR, Hibbert JD, Porter DE, Nichols M, Lamichhane AP, Dabelea D, Mayer-Davis EJ, Standiford D (2010). Evaluating geographic variation in type 1 and type 2 diabetes mellitus incidence in youth in four US regions. Health Place.

[36] Ye J, Wu Y, Yang S, Zhu D, Chen F, Chen J, Ji X, Hou K (2023). The global, regional and national burden of type 2 diabetes mellitus in the past, present and future: A systematic analysis of the global burden of disease study 2019. Front Endocrinol (Lausanne).

[37] de Vegt F, Dekker JM, Jager A, Hienkens E, Kostense PJ, Stehouwer CD, Nijpels G, Bouter LM, Heine RJ (2001). Relation of impaired fasting and postload glucose with incident type 2 diabetes in a Dutch population: The Hoorn study. JAMA.

[38] Dabelea D, Mayer-Davis EJ, Saydah S, Imperatore G, Linder B, Divers J, Bell R, Badaru A, Talton JW, Crume T (2014). Prevalence of type 1 and type 2 diabetes among children and adolescents from 2001 to 2009. JAMA.

[39] Bendinelli B, Palli D, Masala G, Sharp SJ, Schulze MB, Guevara M, van der AD, Sera F, Amiano P (2013). Association between dietary meat consumption and incident type 2 diabetes: The EPIC-InterAct study. Diabetologia.

[40] Romaguera D, Norat T, Wark PA, Vergnaud AC, Schulze MB, van Woudenbergh GJ, Drogan D, Amiano P, Molina-Montes E (2013). Consumption of sweet beverages and type 2 diabetes incidence in European adults: Results from EPIC-InterAct. Diabetologia.

[41] O'Connor LM, Lentjes MAH, Luben RN, Khaw KT, Wareham NJ, Forouhi NG (2014). Dietary dairy product intake and incident type 2 diabetes: A prospective study using dietary data from a 7-day food diary. Diabetologia.

[42] Morris AP, Voight BF, Teslovich TM, Ferreira T, Segrè AV, Steinthorsdottir V, Strawbridge RJ, Khan H, Grallert H, Mahajan A (2012). Large-scale association analysis provides insights into the genetic architecture and pathophysiology of type 2 diabetes. Nat Genet.

[43] Langenberg C, Sharp SJ, Franks PW, Scott RA, Deloukas P, Forouhi NG, Froguel P, Groop LC, Hansen T, Palla L (2014). Gene-lifestyle interaction and type 2 diabetes: The EPIC interact case-cohort study. PLoS Med.

[44] Sebastian MJ, Khan SK, Pappachan JM, Jeeyavudeen MS (2023). Diabetes and cognitive function: An evidence-based current perspective. World J Diabetes.

[45] Sękowski K, Grudziąż-Sękowska J, Pinkas J, Jankowski M (2022). Public knowledge and awareness of diabetes mellitus, its risk factors, complications, and prevention methods among adults in Poland-A 2022 nationwide cross-sectional survey. Front Public Health.

[46] Kupelian V, Link CL, McKinlay JB (2008). Are race/ethnic disparities in the prevalence of nocturia due to socioeconomic status? Results from the Boston area community health (BACH) survey. J Urol.

[47] Sapra A, Bhandari P https://www.ncbi.nlm.nih.gov/books/NBK551501/.

[48] Vieira R, Souto SB, Sánchez-López E, Machado AL, Severino P, Jose S, Santini A, Fortuna A, García ML, Silva AM, Souto EB (2019). Sugar-lowering drugs for type 2 diabetes mellitus and metabolic syndrome-review of classical and new compounds: Part-I. Pharmaceuticals (Basel).

[49] Ramos EL, Dayan CM, Chatenoud L, Sumnik Z, Simmons KM, Szypowska A, Gitelman SE, Knecht LA, Niemoeller E, Tian W (2023). Teplizumab and β-cell function in newly diagnosed type 1 diabetes. N Engl J Med.

[50] Pugh D, Gallacher PJ, Dhaun N (2019). Management of hypertension in chronic kidney disease. Drugs.

[51] Viktor P (2022). Effective treatment of diabetes mellitus and autoimmune diseases by resonance medicine. Int J Med Sci Clin Invent.

[52] Huang YJ, Chen CH, Yang HC (2024). AI-enhanced integration of genetic and medical imaging data for risk assessment of Type 2 diabetes. Nat Commun.

[53] Laakso M, Fernandes Silva L (2022). Genetics of type 2 diabetes: past, present, and future. Nutrients.

[54] Sugandh F, Chandio M, Raveena F, Kumar L, Karishma F, Khuwaja S, Memon UA, Bai K, Kashif M, Varrassi G (2023). Advances in the management of diabetes mellitus: A focus on personalized medicine. Cureus.

[55] Ferreira PL, Morais C, Pimenta R, Ribeiro I, Amorim I, Alves SM, Santiago L (2024). Knowledge about type 2 diabetes: Its impact for future management. Front Public Health.

